# Cardiomyocyte Na^+^ and Ca^2+^ mishandling drives vicious cycle involving CaMKII, ROS, and ryanodine receptors

**DOI:** 10.1007/s00395-021-00900-9

**Published:** 2021-10-14

**Authors:** Bence Hegyi, Risto-Pekka Pölönen, Kim T. Hellgren, Christopher Y. Ko, Kenneth S. Ginsburg, Julie Bossuyt, Mark Mercola, Donald M. Bers

**Affiliations:** 1 Department of Pharmacology, University of California, Davis, 451 Health Sciences Drive, Davis, CA 95616 USA; 2 Cardiovascular Institute and Department of Medicine, Stanford University, Stanford, CA 94305 USA

**Keywords:** Heart failure, Electrophysiology, Calcium, CaMKII, RyR, ROS

## Abstract

Cardiomyocyte Na^+^ and Ca^2+^ mishandling, upregulated Ca^2+^/calmodulin-dependent kinase II (CaMKII), and increased reactive oxygen species (ROS) are characteristics of various heart diseases, including heart failure (HF), long QT (LQT) syndrome, and catecholaminergic polymorphic ventricular tachycardia (CPVT). These changes may form a vicious cycle of positive feedback to promote cardiac dysfunction and arrhythmias. In HF rabbit cardiomyocytes investigated in this study, the inhibition of CaMKII, late Na^+^ current (*I*_NaL_), and leaky ryanodine receptors (RyRs) all attenuated the prolongation and increased short-term variability (STV) of action potential duration (APD), but in age-matched controls these inhibitors had no or minimal effects. In control cardiomyocytes, we enhanced RyR leak (by low [caffeine] plus isoproterenol mimicking CPVT) which markedly increased STV and delayed afterdepolarizations (DADs). These proarrhythmic changes were significantly attenuated by both CaMKII inhibition and mitochondrial ROS scavenging, with a slight synergy with *I*_NaL_ inhibition. Inducing LQT by elevating *I*_NaL_ (by Anemone toxin II, ATX-II) caused markedly prolonged APD, increased STV, and early afterdepolarizations (EADs). Those proarrhythmic ATX-II effects were largely attenuated by mitochondrial ROS scavenging, and partially reduced by inhibition of CaMKII and pathological leaky RyRs using dantrolene. In human induced pluripotent stem cell-derived cardiomyocytes (hiPSC-CMs) bearing LQT3 mutation SCN5A N406K, dantrolene significantly attenuated cell arrhythmias and APD prolongation. Targeting critical components of the Na^+^–Ca^2+^–CaMKII–ROS–*I*_NaL_ arrhythmogenic vicious cycle may exhibit important *on*-target and also *trans*-target effects (e.g., *I*_NaL_ and RyR inhibition can alter *I*_NaL_-mediated LQT3 effects). Incorporating this vicious cycle into therapeutic strategies provides novel integrated insight for treating cardiac arrhythmias and diseases.

## Introduction

**Fig. 1:**
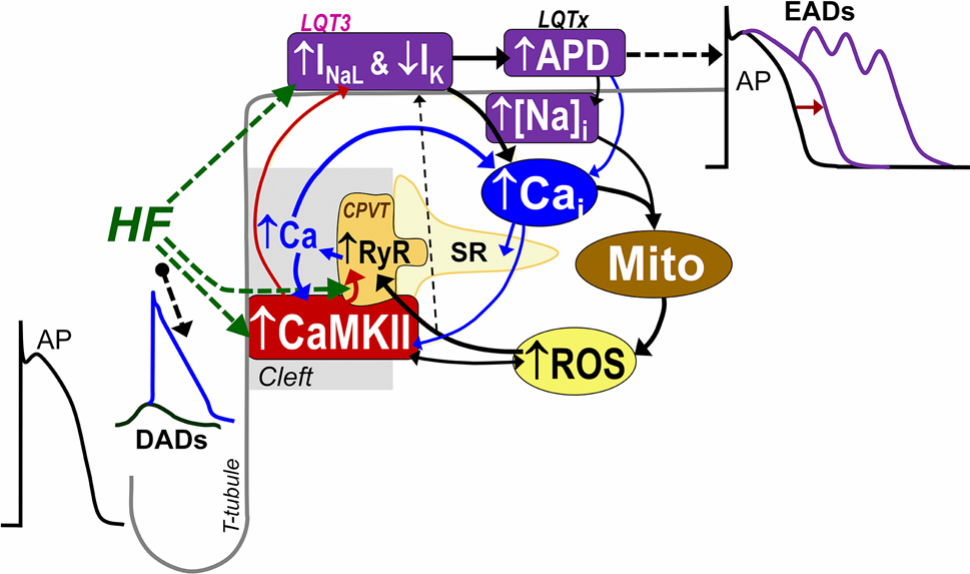
Schematic of arrhythmogenic vicious cycle in heart disease. Heart failure (HF) is characterized by increases of ryanodine receptor (RyR) mediated Ca^2+^ leak, Ca^2+^/calmodulin-dependent kinase II (CaMKII) activity, late Na current (*I*_NaL_), intracellular [Na]_*i*_, action potential duration (APD) and reactive oxygen species (ROS) production, along with reduced repolarization reserve (K^+^ currents, *I*_K_). These factors form a vicious positive feedback cycle that perpetuates HF-associated dysfunction and arrhythmogenesis. For example, the RyR Ca^2+^ leak increases local [Ca^2+^], further activating cleft CaMKII that further enhances RyR leak and *I*_NaL_ (red arrows) and downregulates K^+^ channel expression to reduce *I*_K_, which prolongs APD (as in genetic long QT (LQT) syndromes). Long APDs predispose myocytes to early afterdepolarizations (EADs) and increased intracellular [Na^+^] and [Ca^2+^] loading, which impairs mitochondrial Ca^2+^ handling and may further promote ROS production. ROS can further promote *I*_NaL_ and pathological leaky RyR (as in catecholaminergic polymorphic ventricular tachycardia, CPVT), and increase propensity for delayed afterdepolarizations (DADs). ROS also induces autonomous CaMKII activation closing the positive feedback loop

Heart failure (HF) is characterized by cardiomyocyte Na^+^ and Ca^2+^ dysregulation including elevated intracellular [Na^+^] ([Na^+^]_*i*_) and late Na^+^ current (*I*_NaL_), reduced sarcoplasmic reticulum (SR) Ca^2+^ uptake, and increased diastolic SR Ca^2+^ leak, Na^+^/Ca^2+^ exchange (NCX), and reactive oxygen species (ROS) that contribute to systolic dysfunction and arrhythmias [1, 2, 8, 27, 54]. These alterations also frequently occur in many other heart diseases such as atrial fibrillation [47], ischemia/reperfusion injury [56], hypertrophic cardiomyopathy [6], long QT (LQT) syndromes [48], catecholaminergic polymorphic ventricular tachycardia (CPVT) [32, 70], and diabetes [16, 25]. Moreover, Ca^2+^/calmodulin-dependent protein kinase δ (CaMKIIδ) is also upregulated and chronically active in these diseases [1, 20, 61], and directly promotes I_NaL_ [67] and diastolic SR Ca^2+^ leak through the ryanodine receptor (RyR) [1]. Furthermore, reactive oxygen species (ROS) are increased by CaMKII [49], and elevated [Na^+^]_i_ and intracellular [Ca^2+^] ([Ca^2+^]_*i*_) [7], which in turn further stimulate CaMKII [11] and RyR leak [50]. Thus, these pathological changes in HF are connected via a vicious cycle of positive feedback reinforcing systolic and diastolic dysfunction and arrhythmia mechanisms [18, 45, 68] (see Fig. 1). For example, a primary increase in SR Ca^2+^ leak would promote CaMKII activation, which can promote *I*_NaL_, prolong action potential duration (APD), increase [Na^+^]_*i*_, and ROS production that can further drive the cycle and amplify the functional impacts of initial insults at any given point.

Based on the highly integrative nature of the vicious cycle and the significant impact it has on pathophysiological development, we hypothesized that targeting one of the key components (or combination of those) can prevent cellular proarrhythmia. Inhibition of one component in the vicious cycle with a selective drug is expected to induce the drug-specific *on*-target effect but also *trans*-target effects in the loop as it may reduce the feedback activation of the vicious cycle. The *on*-target drug effects have been the focus of research in recent decades and showed benefits in HF. (1) Selective Na^+^ channel inhibitors (tetrodotoxin, GS-967) were shown to reverse the increased I_NaL_ and the prolongation of the APD in HF [27, 43]. (2) CaMKII inhibition using KN-93 or autocamide-2-related inhibitory peptide (AIP) was shown to reduce RyR leak (for matched SR Ca^2+^ load) in rabbit HF [1], and reduced diastolic Ca^2+^ spark rate in human HF [59]. (3) Mitochondrial-targeted antioxidant MitoTEMPOL normalized global cellular ROS and prevented arrhythmogenic remodelling in a guinea pig model of nonischaemic HF [9]. (4) The pathological leaky conformation of RyR, induced by CaMKII and ROS, can be selectively inhibited using dantrolene, which reduces SR Ca^2+^ leak in CPVT and HF [64]. However, the *trans*-target effects and the strengths of interactions in this vicious cycle have not been systematically investigated.

Here we measured the contribution of the [Na^+^]_*i*_–[Ca^2+^]_*i*_–ROS–CaMKII–RyR leak feedback interactions to proarrhythmic electrophysiological changes in HF rabbits [22]. We also assessed drug-induced RyR leak (mimicking CPVT, [18]) and enhanced I_NaL_ (mimicking long QT3, [24]) in control rabbit cardiomyocytes, and in human induced pluripotent stem cell-derived cardiomyocytes (hiPSC-CMs) carrying arrhythmogenic SCN5A N406K mutation [60].

## Methods

### Rabbit cardiomyocyte isolation

Enzymatic isolation of left ventricular cardiomyocytes from New Zealand White rabbits (male, 3–4-month-old) was performed as previously described [21]. Briefly, animals were injected with heparin (400 U/kg body weight) and anesthetized with isoflurane (3–5%). Hearts were excised and retrograde perfused on constant flow Langendorff apparatus (5 min, 37 °C) with Ca^2+^-free normal Tyrode’s solution, gassed with 100% O_2_. Then, ventricular myocytes were digested using collagenase type II (Worthington) and protease type XIV (Sigma-Aldrich). Ventricular myocytes were dispersed mechanically and filtered through a nylon mesh and allowed to sediment for ~ 10 min. The sedimentation was repeated three times using increasing [Ca^2+^] from 0.125 to 0.25 then 0.5 mmol/L. Finally, ventricular myocytes were kept in Tyrode’s solution at room temperature until use.

### HF rabbit model

HF was induced in New Zealand White rabbits (male, 3–4-month-old) by aortic insufficiency and 4 weeks later by aortic constriction as previously described [22]. Data here reported were obtained from 10 HF and 10 age-matched control rabbits at 2–2.5 years of age. Echocardiography was performed periodically to monitor cardiac function. Cardiomyocytes were isolated from HF rabbits when left ventricular end-systolic dimension exceeded 1.45 cm. HF animals exhibited significant myocardial hypertrophy, enlarged left ventricular dimensions, pulmonary congestion, and abdominal ascites fluid accumulation, similar to our previous studies on this HF rabbit model [22, 54].

### Human iPSC-CMs

Patient specific hiPSC line carrying the SCN5A N406K mutation was generated as previously described [60]. Human iPSC-CMs were differentiated by methods developed in the laboratory of Mark Mercola [44]. At day 20, hiPSC-CMs were placed in a metabolic maturation media and cultured for 5 weeks to improve cardiomyocyte phenotype, including more negative diastolic membrane potentials and Na^+^ current dependent action potentials [12]. Then, hiPSC-CM monolayers were dissociated and re-plated in low density onto Matrigel-coated coverslips 3–5 days before experiments.

### Electrophysiology

Following cell isolation, single cardiomyocytes were transferred to a temperature-controlled chamber (Warner Instruments, Holliston, MA, USA) mounted on a Leica DMI3000 B inverted microscope (Leica Microsystems, Buffalo Grove, IL, USA) and continuously perfused (2 mL/min) with Tyrode’s solution containing (in mmol/L): NaCl 140, KCl 4, CaCl_2_ 1.8, MgCl_2_ 1, HEPES 5, Na-HEPES 5, glucose 5.5; pH = 7.40. Electrodes were fabricated from borosilicate glass (World Precision Instruments, Sarasota, FL, USA) having tip resistances of 2–2.5 MΩ when filled with internal solution containing (in mmol/L): K-aspartate 100, KCl 30, NaCl 8, Mg-ATP 5, phosphocreatine-K_2_ 10, HEPES 10, EGTA 0.01, cAMP 0.002, and calmodulin 0.0001; pH = 7.20 (with KOH). Using this internal solution, the intracellular Ca^2+^ transient and contraction of the cardiomyocyte were preserved [23]. Axopatch 200B amplifier (Axon Instruments Inc., Union City, CA, USA) was used for recordings, and the signals were digitized at 50 kHz by a Digidata 1322A A/D converter (Axon Instruments) under software control (pClamp10.4). The series resistance was typically 3–5 MΩ, and it was compensated by 90%. Experiments were discarded when the series resistance was high or increased by > 10%. Reported voltages are corrected for liquid junction potential. All experiments were conducted at 37 ± 0.1 °C.

Action potentials (APs) were evoked by 2-ms-long supra-threshold depolarizing pulses delivered via the patch pipette. 50 consecutive APs were recorded to examine the average behaviour, and APD at 90% repolarization (APD_90_) was determined. Series of 50 consecutive APs were analysed to estimate short-term variability of APD_90_ (STV) according to the following formula: STV = Σ(│APD_*n*+1_–APD_*n*_│)/[(*n*_beats_−1)×√2], where APD_*n*_ and APD_*n*+1_ indicate the durations of the *n*th and (*n* + 1)th APs, and n_beats_ denotes the total number of consecutive beats analysed. APD alternans magnitude was calculated as the difference between the average APD_90_ of odd and even numbered beats during 50 consecutive APs recorded. Diastolic arrhythmogenic activities were elicited by cessation of 1-min tachypacing, and membrane potential was recorded for additional 1 min. Delayed afterdepolarizations (DADs) were defined as > 1 mV depolarization within 0.5 s. Spontaneous APs (sAPs) were defined as depolarizations showing overshoot with a fast upstroke phase. Early afterdepolarizations (EADs) were assessed at 0.2 Hz pacing, and EADs were defined as > 3 mV depolarization during AP repolarization.

AP-clamp experiments were performed to measure I_NaL_ as previously described [19]. A typical rabbit AP was used to AP-clamp cells at 2 Hz pacing frequency. I_NaL_ was measured as GS-967 (1 μmol/L)-sensitive current in control and following enhancement with ATX-II (5 nmol/L).

Cell pretreatments with MitoTEMPOL and AIP (myristoylated) started 30 min before the experiments, and the drugs were also added to both the perfusion and pipette solutions.

Chemicals and reagents were purchased from Sigma-Aldrich (St. Louis, MO, USA), if not specified otherwise. ATX-II and MitoTEMPOL were from Abcam (Cambridge, MA, USA), and GS-967 was from Cayman Chemical (Ann Arbor, MI, USA).

### Calcium imaging

To measure SR Ca^2+^ concentration ([Ca^2+^]_SR_), freshly isolated rabbit cardiomyocytes were loaded with 8 μmol/L Mag-Fluo-4-AM (Invitrogen, Carlsbad, CA, USA) with 0.2% Pluronic F-127 (Biotium, Hayward, CA, USA) for 2 h at room temperature. Subsequently, cells were washed twice in fresh Tyrode’s solution for 30 min to allow de-esterification to occur. Then, cardiomyocytes were placed in a narrow bath chamber with embedded field stimulation electrodes (RC-27NE2, Warner Instruments) and stimulated at 0.5 Hz frequency in Tyrode’s solution at room temperature (22 ± 1 °C). Mag-Fluo-4 was excited at 480 nm wavelength using an Optoscan monochromator (Cairn Research, Faversham, UK) and fluorescence emission was collected at 535 ± 15 nm.

To measure [Ca^2+^]_*i*_, cardiomyocytes were loaded with 10 μmol/L Rhod2-AM (ThermoFisher, Waltham, MA, USA) for 10 min at room temperature and subsequently left to de-esterify in fresh Tyrode’s solution for a minimum of 30 min. Then, cardiomyocytes were placed in a RC-27NE2 recording chamber and stimulated at 0.5 Hz frequency in Tyrode’s solution at room temperature. Rhod2 was excited at 561 nm wavelength using an Optoscan monochromator, and fluorescence was collected at 530 ± 20 nm. Fluorescence signals were recorded after steady state was reached in the cell during pacing.

### Statistical analysis

Data are presented as Mean ± SEM. Statistical significance of differences for normally distributed data was tested by paired Student’s *t*-test to compare two groups and ANOVA with Dunnett’s or Tukey’s post-hoc test to compare multiple groups. For non-normally distributed data, we used Wilcoxon matched-pairs signed rank test, Mann–Whitney test, and Kruskal–Wallis ANOVA with Dunn’s post-hoc test. Differences were deemed significant if *P* < 0.05.

## Results

### Enhanced RyR leak, CaMKII, and ***I***_NaL_ all contribute to arrhythmogenic AP changes in HF

**Fig. 2:**
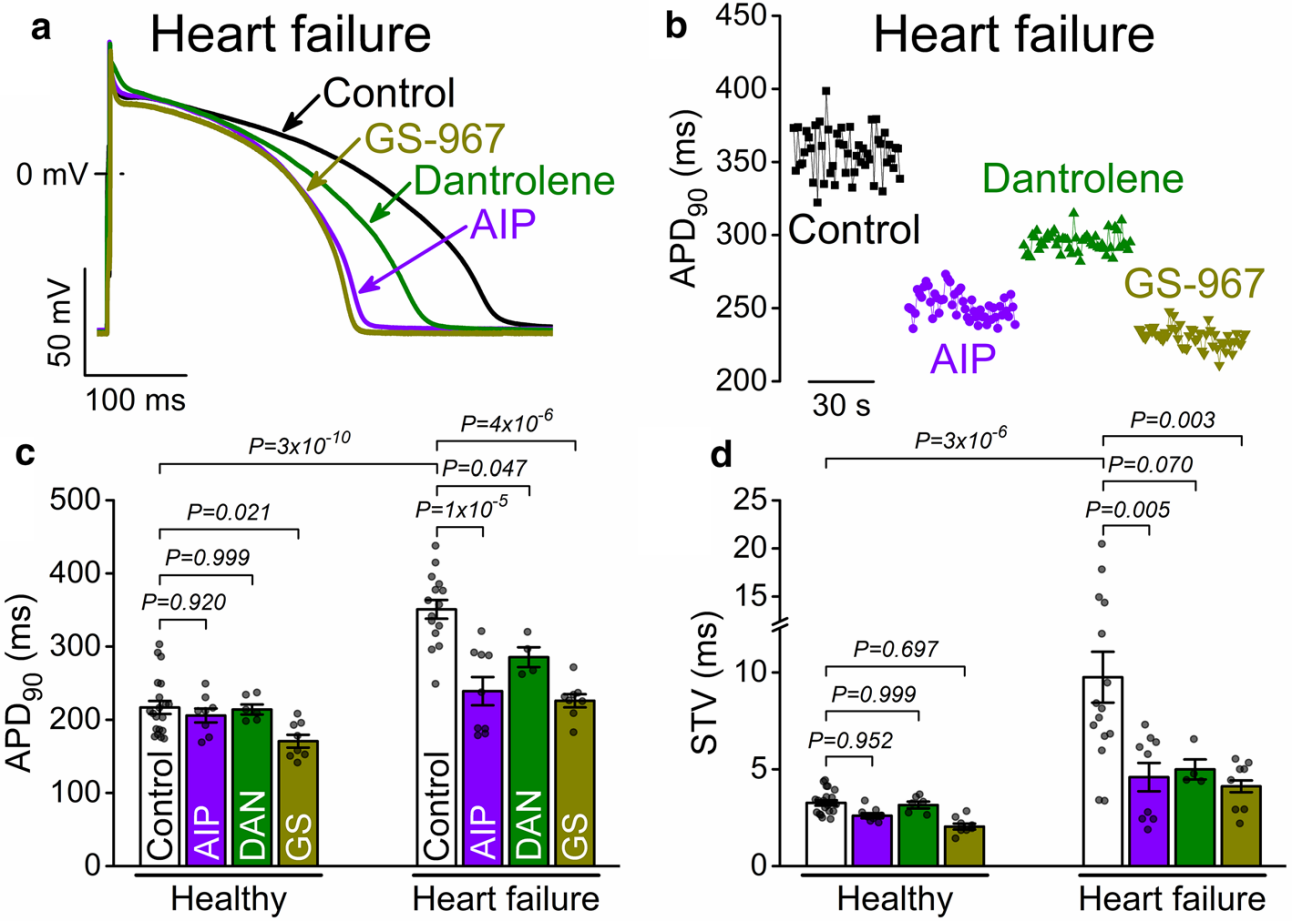
CaMKII, leaky RyRs, and late Na^+^ current promote arrhythmogenic AP changes in HF. **a** Representative APs in failing rabbit ventricular myocytes in control and following treatments with either the selective CaMKII inhibitor AIP (1 μmol/L), the pathological RyR conformation inhibitor dantrolene (DAN, 10 μmol/L) or the late Na^+^ current inhibitor GS-967 (GS, 1 μmol/L) **b** 50 consecutive AP durations at 90% repolarization (APD_90_). **c** APD_90_ in age-matched healthy and failing rabbit cardiomyocytes. ANOVA with Dunnett’s multiple comparisons test. **d** Short-term variability (STV) of APD_90_. Cells were paced at 1 Hz. ANOVA with Dunnett’s multiple comparisons test. (*N* = 10 HF and 10 age-matched control rabbits, each individual myocyte (*n*) is shown as a data point.)

Cardiomyocytes in our HF rabbit model exhibited significantly prolonged APD_90_ and greater short-term APD variability (STV) vs. age-matched healthy controls at 1 Hz at 37 °C (Fig. 2a–d). We tested the effects of specific inhibition of either CaMKII, I_NaL_ or RyR leak on APs of rabbit ventricular myocytes isolated from failing and healthy hearts. Pretreatment with the selective CaMKII inhibitor peptide AIP (1 μmol/L) or the late Na^+^ current inhibitor GS-967 (1 μmol/L) significantly shortened APD_90_ and reduced STV in failing myocytes to the level of healthy age-matched myocytes (Fig. 2a–d). Interestingly, the pathological RyR conformation inhibitor dantrolene (10 μmol/L) also shortened APD_90_ and reduced STV in HF (Fig. 2a–d). Importantly, in healthy control myocytes neither AIP nor dantrolene had significant effects on APD_90_, and GS-967 only slightly shortened APD_90_ in healthy myocytes (Fig. 2c). In healthy myocytes GS-967 and AIP slightly reduced STV, but those differences were quantitatively small compared to those for HF myocytes (Fig. 2d). The effects of direct I_NaL_ inhibition (GS-967) and CaMKII (AIP) on APD and STV could be expected because *I*_NaL_ and CaMKII activity are known to be elevated in HF, and CaMKII has been shown to directly enhance *I*_NaL_ [5, 27, 67]. However, the potent effect of dantrolene on APD and STV is evidence that the pathological RyR state in HF increases APD and STV, which may be mediated by the vicious cycle via SR Ca^2+^ leak-promoted CaMKII and *I*_NaL_.

### RyR leak increases APD-variability via mitoROS-CaMKII-***I***_NaL_ feedback

**Fig. 3:**
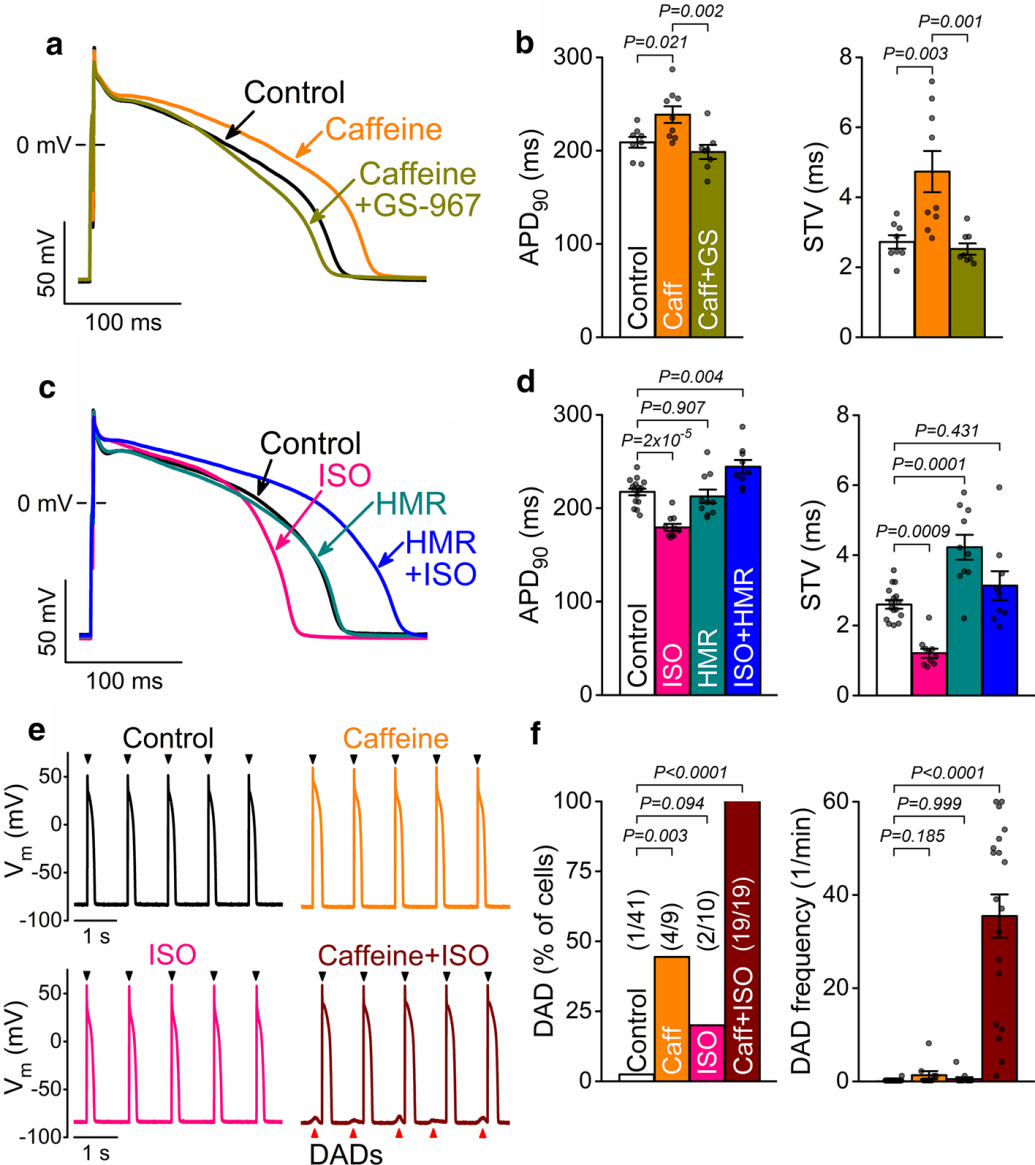
Caffeine and isoproterenol induced AP changes in healthy rabbit ventricular myocytes. **a** Representative rabbit ventricular APs in control and low-dose caffeine (Caff, 200 μmol/L), and after application of the late Na^+^ current inhibitor GS-967 (GS, 1 μmol/L). **b** APD_90_ and STV were increased by Caff, then restored by GS. **c** Representative APs in control and following β-adrenergic agonist isoproterenol (ISO, 100 nmol/L) stimulation, and after application of the slow delayed rectifier K^+^ current inhibitor HMR-1556 (HMR, 1 μmol/L). **d** APD_90_ and its short-term variability (STV) in control, ISO, HMR, and ISO + HMR. **e** APs and delayed afterdepolarizations (DADs, indicated by red arrowheads) following combined treatment with Caff + ISO at 1 Hz steady-state pacing. **f** Percent of cells showing DADs and the frequency of DADs during 1 Hz pacing for 1 min. APD_90_ and STV were compared using ANOVA with Tukey’s multiple comparisons test. Number of cells showing DADs was compared using Fisher’s exact test. DAD frequencies were compared using Kruskal–Wallis ANOVA with Dunn’s multiple comparisons test. (*N* = 5–17 animals in each treatment group, each individual myocyte (*n*) is shown as a data point.)

To separate RyR leak from the complex HF phenotype, in terms of the arrhythmogenic feedback signalling network (i.e., the vicious cycle), we induced RyR leak by low [caffeine] (200 μmol/L) and isoproterenol (ISO; 100 nmol/L) in healthy rabbit ventricular myocytes. Caffeine (3 min) slightly prolonged APD_90_ and increased STV (Fig. 3a, b). The additional application of GS-967 decreased APD_90_ and STV back to control suggesting a role for Ca^2+^-dependent upregulation of I_NaL_ (Fig. 3a, b). In contrast to caffeine effects, ISO (3 min) shortened APD_90_ and reduced STV (Fig. 3c, d). Then, inhibition of the slow delayed rectifier K^+^ current (I_Ks_) using HMR-1556 (HMR, 1 μmol/L) in the presence of ISO markedly prolonged APD_90_ (mimicking LQT1) and significantly increased STV, while HMR had no effect on APD_90_ at basal conditions without ISO stimulation (Fig. 3c, d). These data suggest that the upregulation of I_Ks_ counterbalances the increased I_NaL_ during β-adrenergic stimulation. Nonetheless, during steady-state pacing at 1 Hz, only very few DADs occurred in a small fraction of cells treated with either caffeine or ISO alone (Fig. 3e, f). However, when caffeine and ISO were applied together, several DADs were observed in every cell measured (Fig. 3e, f). This suggests that the increased SR Ca^2+^ leak (caffeine) must be combined with enhanced SR Ca^2+^ loading (ISO) to induce DADs in healthy myocytes. Hence, in the following, we used a combination treatment of low [caffeine] and ISO to investigate the role of the vicious cycle in proarrhythmic AP changes.

**Fig. 4:**
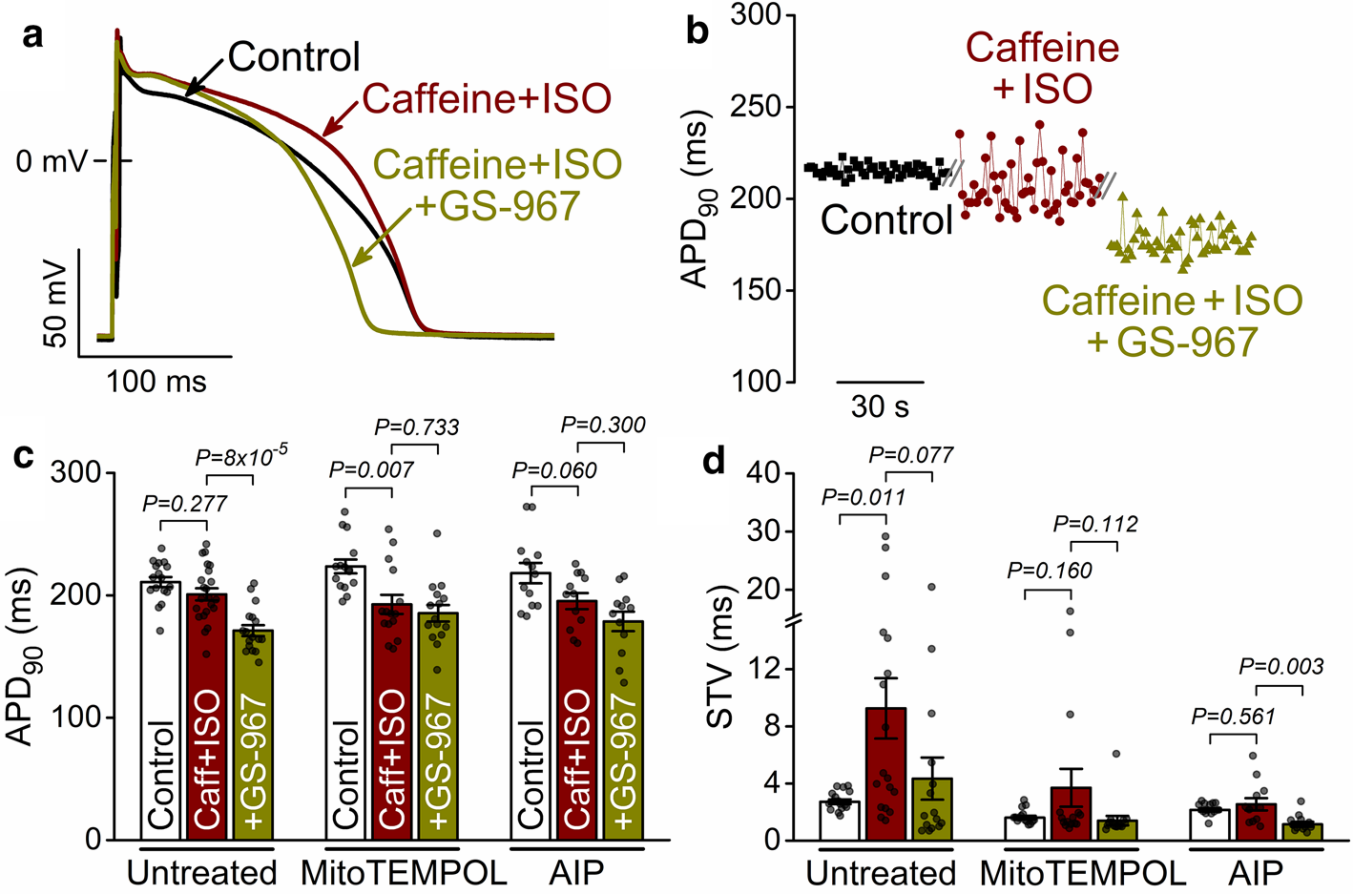
RyR leak enhances late Na^+^ current, mito-ROS and CaMKII to induce proarrhythmic AP changes. **a** Representative rabbit ventricular APs in control and following caffeine (Caff, 200 μmol/L) and isoproterenol (ISO, 100 nmol/L) stimulation, and after application of the late Na^+^ current inhibitor GS-967 (1 μmol/L). **b** Fifty consecutive APD_90_ over time demonstrating increased APD_90_-variability following application of caffeine and isoproterenol. **c** APD_90_ in cells without pretreatment and following pretreatment with MitoTEMPOL (20 μmol/L) and CaMKII inhibitor AIP (1 μmol/L). **d** Short-term variability (STV) of APD_90_. ANOVA with Tukey’s multiple comparisons test. (*N* = 6–10 animals in each treatment group, each individual myocyte (*n*) is shown as a data point.)

Following 3-min caffeine + ISO treatment, the AP plateau was significantly elevated (Fig. 4a), but the APD_90_ did not change (Fig. 4a, c); however, STV was markedly increased (Fig. 4b, d). Importantly, the I_NaL_ inhibitor GS-967 (and not a direct SR Ca^2+^ leak modulator) significantly shortened APD_90_ following caffeine + ISO and attenuated the increase in STV (Fig. 4a–d). Cell pretreatment with the mitochondrial ROS (mitoROS) scavenger mitoTEMPOL (20 μmol/L) or AIP did not change significantly baseline APD_90_ and STV (Fig. 4c, d). However, caffeine + ISO induced APD_90_ shortening in both mitoTEMPOL and AIP pretreated cells (Fig. 4a–d). The additional application of GS-967 no longer altered APD_90_ in mitoTEMPOL and AIP pretreated cells (Fig. 4a–d). These data indicate that mitoROS-CaMKII signalling markedly upregulates *I*_NaL_ following RyR leak enhancement and contributes to increased beat-to-beat APD_90_-variability. Thus, SR Ca^2+^ leak enhancement recruits ROS, *I*_NaL_ and CaMKII as part of its integrated response.

### RyR leak-induced DADs are suppressed by synergistic inhibition of ***I***_NaL_, mitoROS and CaMKII

**Fig. 5:**
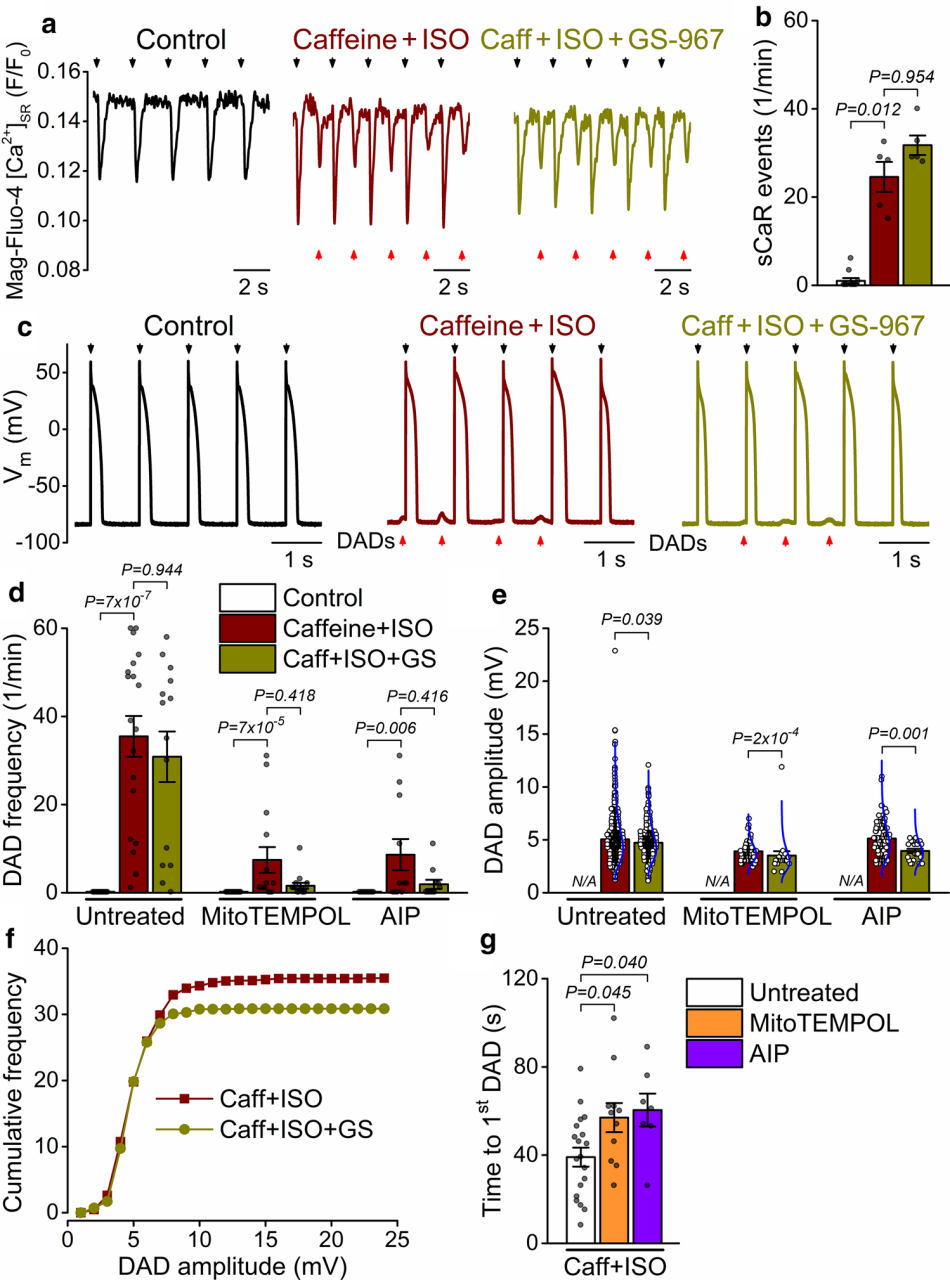
RyR leak synergizes with mito-ROS, CaMKII, and late Na^+^ current to promote DADs. **a** Sarcoplasmic reticulum Ca^2+^ release (monitored as Mag-Fluo-4 fluorescence) in control and following caffeine (Caff, 200 μmol/L) and isoproterenol (ISO, 100 nmol/L) stimulation, and after application of GS-967 (GS, 1 μmol/L). Rabbit ventricular cells were paced at 0.5 Hz steady state. Black arrowheads indicate pacing signals, and red arrowheads indicate spontaneous Ca^2+^ release (sCaR) events. **b** Frequency of sCaR events. **c** APs and delayed afterdepolarizations (DADs, indicated by red arrowheads) at 1 Hz steady-state pacing. **d** DAD frequency during 1 Hz pacing for 1 min in cells without pretreatment and following pretreatment with MitoTEMPOL (20 μmol/L) and CaMKII inhibitor AIP (1 μmol/L). **e** DAD amplitude. Blue lines represent lognormal distribution curves. **f** Cumulative DAD frequencies as a function of DAD amplitudes. **g** Time to first DAD after application of caff + ISO. Cells were paced at 1 Hz steady-state. DAD and sCaR frequencies were compared using Kruskal–Wallis ANOVA with Dunn’s multiple comparisons test; DAD amplitudes were compared using Mann–Whitney test. (*N* = 6–9 animals in each treatment group, each individual myocyte (*n*) is shown as a data point.)

Because RyR leak is associated with the development of arrhythmogenic delayed afterdepolarizations (DADs), we tested the contribution of enhanced I_NaL_-mitoROS-CaMKII feedback to DAD occurrence. Caffeine + ISO induced spontaneous SR Ca^2+^ release (sCaR) events between paced beats in myocytes loaded with the intra-SR [Ca^2+^] ([Ca^2+^]_SR_) fluorescent indicator, Mag-Fluo-4 (Fig. 5a, b). In parallel current-clamp experiments, caffeine + ISO also induced DADs with a frequency of 35 ± 5/min and amplitude of 5.1 ± 0.2 mV during steady-state pacing (Fig. 5c–e). Following GS-967 treatment, the frequency of sCaRs (Fig. 5b) and DADs (Fig. 5d) was unchanged, but GS-967 significantly reduced the DAD amplitude (Fig. 5e), especially the large DADs (Fig. 5f). Importantly, DAD frequency was significantly reduced in cells preincubated with either mitoTEMPOL or AIP (7 ± 3/min and 9 ± 4/min, respectively; Fig. 5d). MitoTEMPOL and AIP also significantly increased DAD latency upon caffeine + ISO treatment (Fig. 5g). Moreover, cumulative application of GS-967 tended to further reduce DAD frequency in mitoTEMPOL and AIP pretreated cells (2 ± 1/min in both cases; Fig. 5d).

**Fig. 6:**
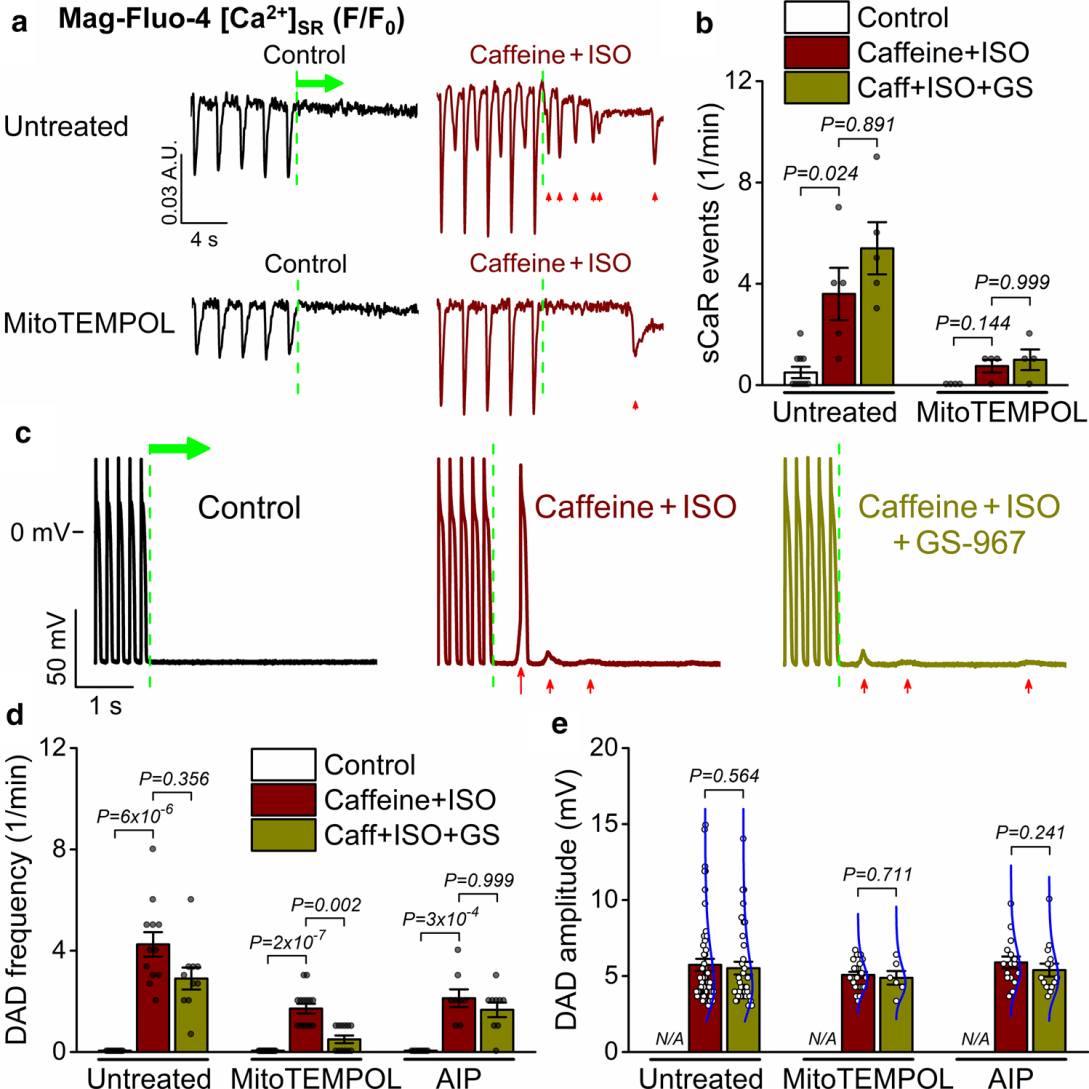
Mito-ROS, CaMKII and late Na^+^ current promote spontaneous diastolic activities. **a** Spontaneous SR Ca^2+^ release (sCaR) events following cessation of pacing (indicated by green dashed lines) were induced by caffeine (Caff, 200 μmol/L) and isoproterenol (ISO, 100 nmol/L) stimulation. Pretreatment with MitoTEMPOL (20 μmol/L) attenuated the sCaR events. Rabbit ventricular myocytes were loaded with Mag-Fluo-4AM. **b** Frequency of sCaR events. **c** Delayed afterdepolarizations (DADs) following cessation of tachypacing (5 Hz), which elicited spontaneous APs in some instances. **d**, **e** DAD frequency and amplitude during a 1-min recording following cessation of tachypacing. Late Na^+^ current was inhibited using GS-967 (GS, 1 μmol/L), and CaMKII was inhibited using AIP (1 μmol/L). Blue lines represent lognormal distribution curves. DAD and sCaR frequencies were compared using Kruskal–Wallis one-way ANOVA with Dunn’s multiple comparisons test; DAD amplitudes were compared using Mann–Whitney test. (*N* = 6–7 animals in each treatment group, each individual myocyte (*n*) is shown as a data point.)

Next, we examined the stability of the SR Ca^2+^ release system following cessation of pacing. Caffeine + ISO induced spontaneous SR Ca^2+^ release events, which were attenuated in mitoTEMPOL-treated cells (Fig. 6a, b). Caffeine + ISO induced multiple DADs and, in a few instances, spontaneous APs (sAPs) following cessation of tachypacing (Fig. 6c). The frequency of DADs was markedly attenuated by cell pretreatment with mitoTEMPOL or AIP (Fig. 6d). Addition of GS-967 reduced DAD frequency only in MitoTEMPOL-pretreated cells but did not change DAD amplitude following cessation of pacing (Fig. 6e).

These data indicate that mitochondrial superoxide production and CaMKII activation markedly enhance DADs in cells with pronounced RyR leak, and a combined treatment of mitoTEMPOL or AIP and GS-967 is largely protective against DADs.

### Enhanced ***I***_NaL_ induces RyR leak that further prolongs APD

**Fig. 7:**
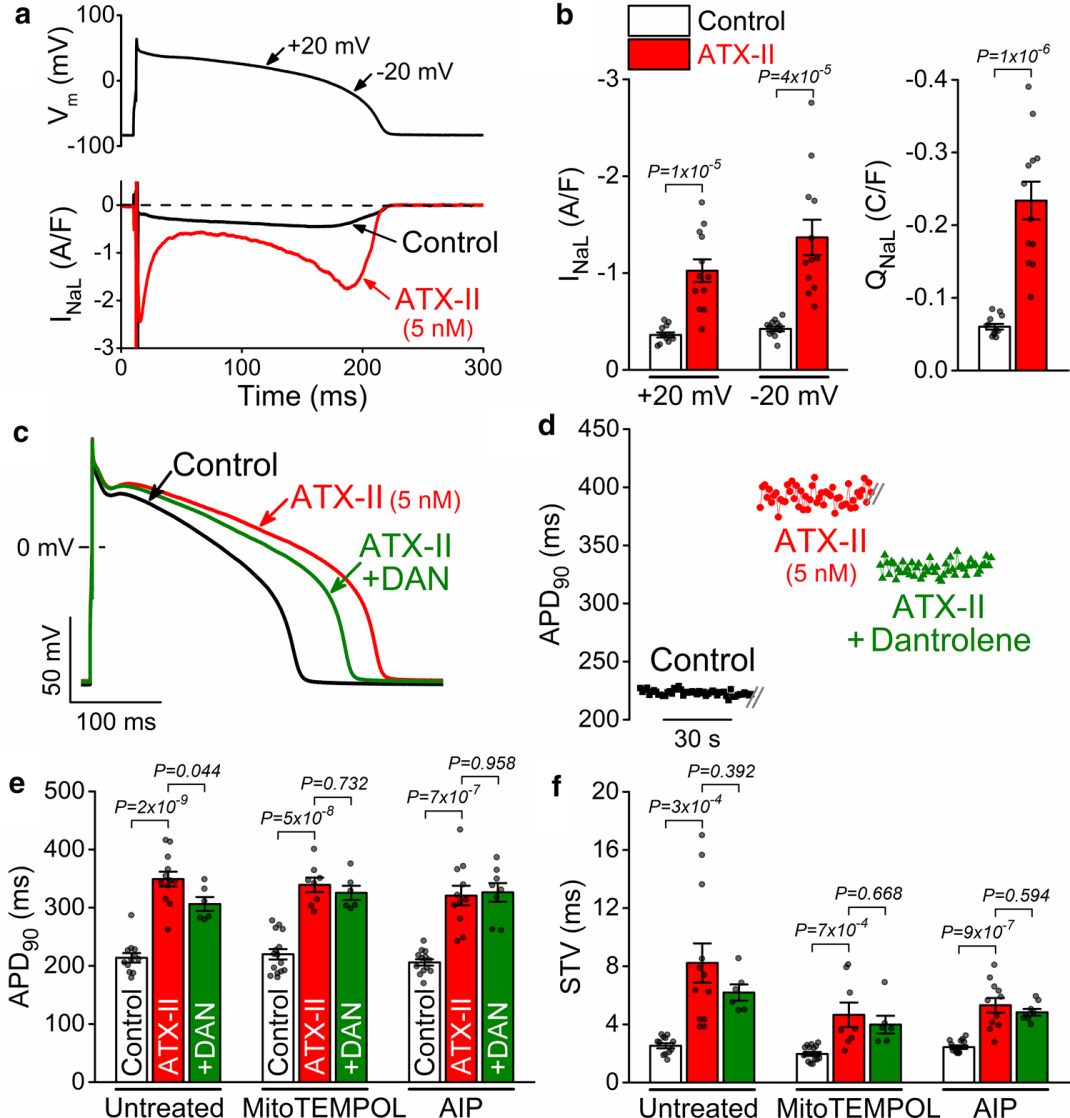
Dantrolene attenuates APD prolongation induced by enhanced late Na^+^ current. **a** Late Na^+^ current (*I*_NaL_) in AP-clamped rabbit ventricular myocytes in control and following ATX-II (5 nmol/L). **b** *I*_NaL_ density at + 20 mV and − 20 mV, and net charge (*Q*_NaL_) carried by *I*_NaL_ under AP-clamp. Student’s *t*-test. **c** Representative rabbit ventricular APs in control and in the presence of ATX-II, and after application of dantrolene (DAN, 10 μmol/L). **d** Increased APD_90_-variability following ATX-II. **e** APD_90_ in cells without pretreatment and following pretreatment with MitoTEMPOL (20 μmol/L) and CaMKII inhibitor AIP (1 μmol/L). **f** Short-term variability (STV) of APD_90_. ANOVA with Tukey’s multiple comparisons test. (*N* = 5–7 animals in each treatment group, each individual myocyte (*n*) is shown as a data point.)

Next, we tested whether proarrhythmic AP changes induced by enhanced I_NaL_ are attenuated by dantrolene in healthy rabbit ventricular myocytes. Anemone toxin II (ATX-II, 5 nmol/L) significantly enhanced *I*_NaL_ during AP-clamp, and the net charge carried by I_NaL_ increased by 3.9-fold (Fig. 7a, b). In current-clamp, ATX-II also prolonged APD_90_ (mimicking LQT3) and markedly increased STV (Fig. 7c, d). Importantly, dantrolene attenuated the ATX-II-induced APD prolongation (Fig. 7c), and this dantrolene effect was absent in cells preincubated with mitoTEMPOL and AIP (Fig. 7e). Moreover, mitoTEMPOL and AIP slightly reduced the increase in STV by ATX-II (Fig. 7f). These data indicate that the mitoROS-CaMKII-induced RyR leak contributes to APD prolongation when I_NaL_ is enhanced by ATX-II. However, the dantrolene impact on APD prolongation is modest.

### ***I***_NaL_ induced EADs are attenuated by dantrolene, mitoTEMPOL and AIP

**Fig. 8:**
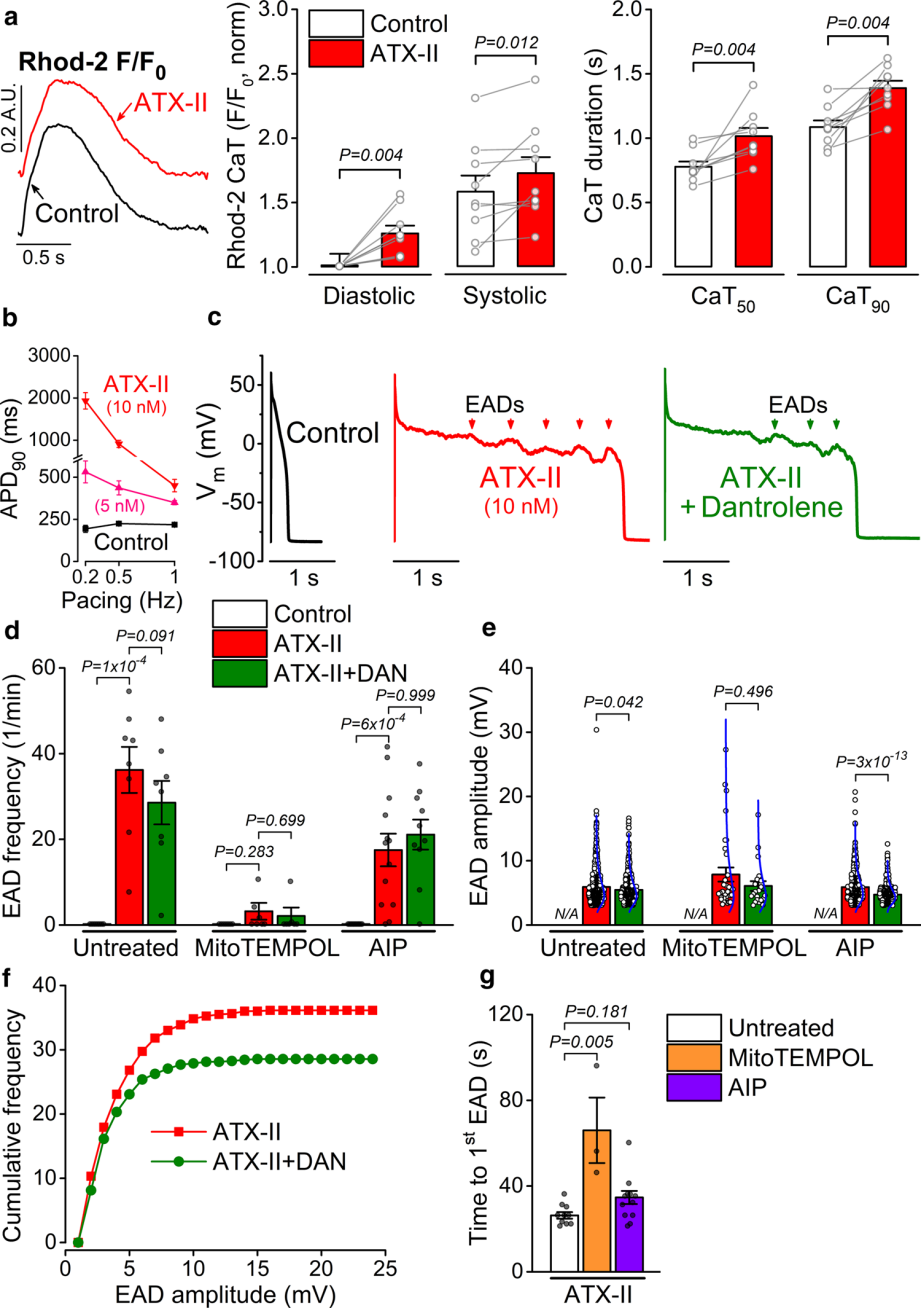
Enhanced late Na^+^ current induces RyR leak, mito-ROS and CaMKII to promote EADs. **a** Intracellular Ca^2+^ transient (CaT) measured as Rhod-2 fluorescence in control and ATX-II (10 nmol/L) in rabbit ventricular myocytes paced at 0.5 Hz. The error bar on the control diastolic value indicates the degree of variability in the baseline raw *F* (*F*/non-cellular background), and *F*_0_ is the control baseline *F* in each cell. Wilcoxon matched-pairs signed rank test. **b** Reverse-rate dependent APD_90_ prolongation by ATX-II (5 and 10 nmol/L). **c** Early afterdepolarizations (EADs, red arrowheads) at 0.2 Hz steady-state pacing in a representative rabbit ventricular cell in control and ATX-II (10 nmol/L), and after application of dantrolene (DAN, 10 μmol/L). **d**, **e** EAD frequency and amplitude during pacing in cells without pretreatment and following pretreatment with MitoTEMPOL (20 μmol/L) and CaMKII inhibitor AIP (1 μmol/L). Blue lines represent lognormal distribution curves. **f** Cumulative EAD frequencies as a function of EAD amplitudes. **g** Time to first EAD after application of ATX-II. Cells were paced at 0.2 Hz steady-state. EAD frequencies were compared using Friedman repeated measure ANOVA with Dunn’s multiple comparisons test. EAD amplitudes were compared using Mann–Whitney test. (*N* = 3–7 animals in each treatment group, each individual myocyte (*n*) is shown as a data point.)

ATX-II also increased systolic and diastolic intracellular Ca^2+^ levels (measured as change in Rhod-2 fluorescence) and prolonged the [Ca^2+^]_i_ transient (CaT, Fig. 8a). ATX-II markedly prolonged APD at low pacing rates (Fig. 8b) and 10 nmol/L ATX-II induced early afterdepolarizations (EADs) (Fig. 8c). Dantrolene slightly attenuated both the frequency and amplitude of EADs in ATX-II (Fig. 8c–f). Interestingly, mitoTEMPOL preincubation markedly reduced the frequency (Fig. 8d) but not the amplitude of the EADs (Fig. 8e); however, mitoTEMPOL delayed the time to the first EAD significantly (Fig. 8g). In contrast to mitoTEMPOL, AIP was only slightly protective against EAD formation induced by ATX-II (Fig. 8d). These data indicate that ROS production, and also slightly CaMKII and RyR leak contribute to ATX-II induced EADs.

### Dantrolene reduces arrhythmogenic activities in SCN5A N406K hiPSC-CMs

Next, we tested the effects of dantrolene in hiPSC-CMs carrying the SCN5A N406K LQT3 mutation, which has been associated with significant QT prolongation, increased risk of torsade de pointes-type ventricular tachycardia and sudden cardiac death [60]. Previous biophysical characterization [31] showed that the mutant channels exhibit an interesting mixed phenotype with increased I_NaL_ as gain-of-function (long QT3) and a decreased peak I_Na_ (due to reduced surface expression of Na^+^ channels) as loss-of-function (Brugada syndrome). Importantly, these changes in Na^+^ channel function are similar to the CaMKII-mediated effects [67] and remodelling in HF [65]. Moreover, hiPSC-CMs carrying the SCN5A N406K mutation also showed impaired intracellular Ca^2+^ handling and Ca^2+^-dependent arrhythmias [60].

**Fig. 9:**
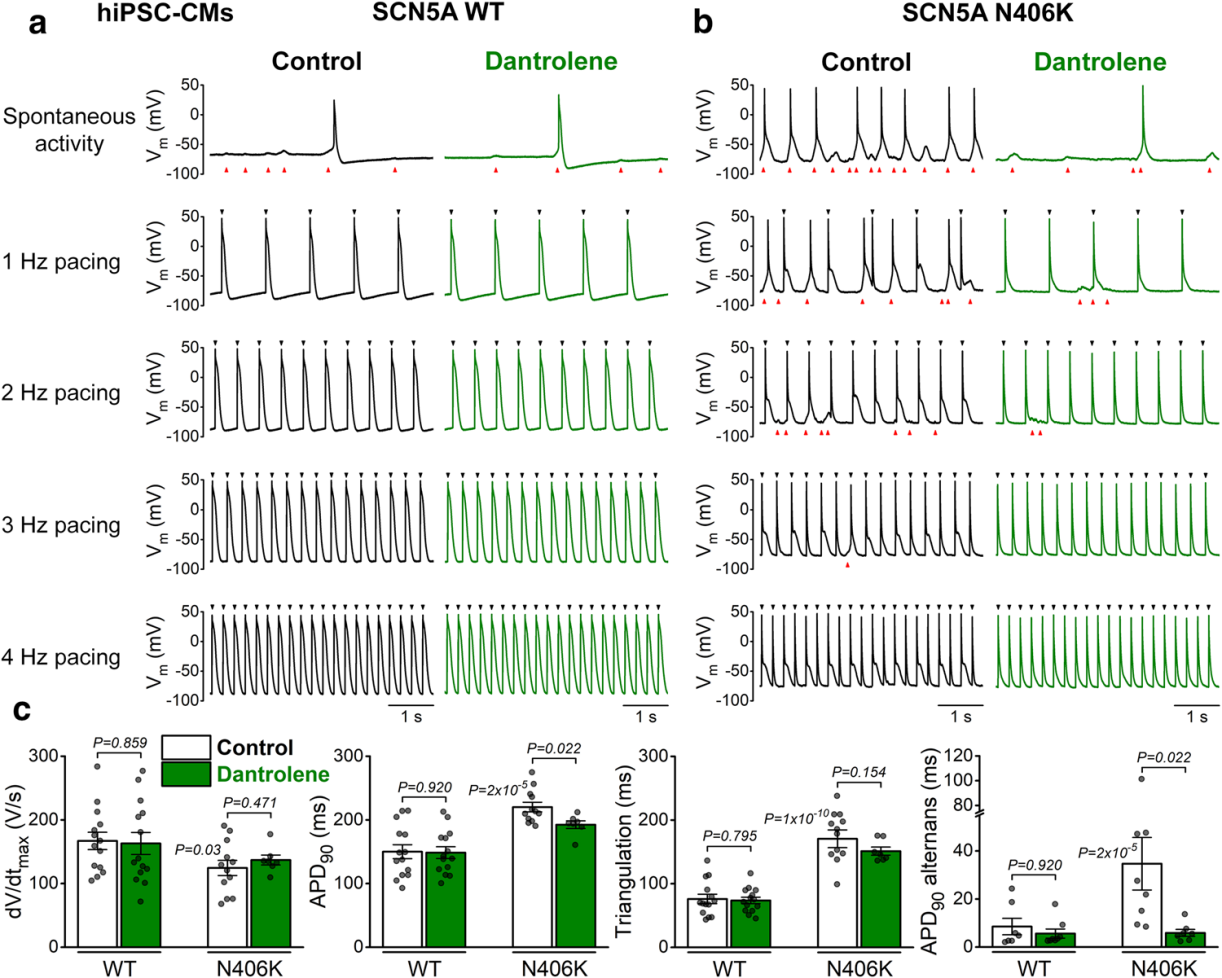
Dantrolene reduces arrhythmogenic activities in SCN5A N406K hiPSC-CMs. **a** Series of action potentials (APs) without pacing and using increasing pacing frequencies from 1 to 4 Hz in control and following dantrolene (10 μmol/L) treatment in a representative wild-type (WT) hiPSC-CM. Black arrowheads on top of each trace indicate pacing signals. Red arrowheads at the bottom of each trace indicate spontaneous depolarizations. **b** Representative APs in control and following dantrolene treatment in SCN5A N406K hiPSC-CM. **c** Summary data on maximal upstroke velocity (d*V*/d*t*_max_), AP duration at 90% repolarization (APD_90_), AP triangulation (APD_90_–APD_50_) at 1 Hz pacing, and the magnitude of APD_90_ alternans in subsequent beats at 4 Hz pacing. Student’s paired *t*-test and ANOVA with Tukey’s multiple comparisons test. (Each individual hiPSC-CM (*n*) is shown as a data point.)

APs in SCN5A N406K and wild type (WT) hiPSC-CMs, cultured in a metabolic maturation media and paced at 1 Hz [12], exhibited sufficiently negative diastolic *V*_m_ to enable robust Na^+^ channel availability and AP rate of rise (Fig. 9a–c). Even so the N406K vs. WT cells exhibited lower maximal upstroke velocity (d*V*/d*t*_max_), prolonged APD_90_, and significant AP triangulation, in line with data in literature and the expected consequences of decreased peak I_Na_ and increased *I*_NaL_ (Fig. 9a–c). Cells carrying the N406K mutation also had frequent spontaneous depolarizations (Fig. 9b). Moreover, significant APD alternans occurred in N406K mutants at higher pacing rates (starting at 3 Hz; Fig. 9b). Importantly, dantrolene treatment significantly reduced the spontaneous depolarizations and shortened APD_90_ in N406K, while it had no effect on APD_90_ in WT hiPSC-CMs (Fig. 9a–c). Dantrolene also increased the APD alternans threshold frequency (from 3 to 4 Hz) and reduced the amplitude of APD alternans (Fig. 9c). These data reinforce the suggested interplay between *I*_NaL_ and RyR in the vicious cycle.

## Discussion

Impairments in cardiomyocyte Na^+^ and Ca^2+^ handling are characteristic of HF and contribute to contractile dysfunction and arrhythmias [45, 54, 68]. In our HF rabbit model, [Na^+^]_*i*_ was found to be 3 mmol/L higher than in control [8]. In agreement with this, *I*_NaL_ was increased by 82% in failing rabbit myocytes, and the *I*_NaL_ upregulation was predominantly CaMKII-dependent [27]. CaMKIIδ_C_ expression and autophosphorylation were increased by 112% and 260%, respectively, in HF rabbit hearts, and similar increases were found in human heart samples from patients with dilated and ischemic cardiomyopathies [5]. CaMKII-dependent phosphorylation of RyR2 at S2814 was increased by 105% in rabbit HF [1] and led to increased SR Ca^2+^ leak at a given SR Ca^2+^ load [58]. Moreover, NCX expression and NCX current were also increased by 93% and 120%, respectively, in HF rabbits [53]. Furthermore, the membrane resistance is increased in HF due to 25–50% reduction in inward rectifier K^+^ current (*I*_K1_) [22, 54], thus, a given depolarizing current can cause larger DADs. The magnitude of I_K1_ reduction quantitatively matches the downregulation of Kcnj2/K_ir_2.1 expression upon chronic CaMKII overexpression [21]. Taken together, less Δ[Ca^2+^]_i_ is required to trigger a spontaneous AP in failing cardiomyocytes [54]. Importantly, CaMKII inhibition was shown to prevent DADs in isolated failing cardiomyocytes [22] and reduced in vivo arrhythmia inducibility in HF [28]. Calcium- and CaMKII dependent arrhythmias were also demonstrated in long QT caused by either gain-of-function mutation in Na^+^ channels [72] or loss-of-function mutation in K^+^ channels [63]. Along the same lines, in RyR-mutant CPVT, inhibition of CaMKII markedly attenuated proarrhythmic activities [4, 38]. These data indicate the activation of the vicious cycle and its pivotal role in arrhythmogenesis in HF, LQT, and CPVT.

ROS is a critical mediator of pathological cellular remodelling and contributes to impaired cardiomyocyte Na^+^ and Ca^2+^ homeostasis in heart diseases [15]. Our data support the concept of a strong, bidirectional feedback between SR Ca^2+^ leak and increased ROS [17]. ROS can oxidize RyRs [50] and induce autonomous CaMKII activation [11], both further increase SR Ca^2+^ leak [62, 69]. SR Ca^2+^ leak then may increase Ca^2+^ uptake into neighbouring mitochondria via the mitochondrial Ca^2+^ uniporter (MCU) [3, 36]. Oxidation of MCU can also increase its activity [10]. Furthermore, CaMKII can also increase ROS via NADPH oxidase 2 (NOX2) [41, 49]. While some data in isolated mitochondria suggested elevated mitochondrial [Ca^2+^] in HF (due to leaky RyRs and increased mitochondrial Ca^2+^ uptake [57]), more direct HF measurements in intact guinea-pig ventricular myocytes indicated reduced mitochondrial [Ca^2+^] (due to elevated [Na^+^]_*i*_, lower CaTs and greater Ca^2+^ extrusion via mitochondrial Na^+^/Ca^2+^ exchange) [42]. Moreover, both increased and decreased mitochondrial [Ca^2+^] may increase ROS production [7]. Interestingly, a recent paper showed that moderate overexpression of MCU that enhances mitochondrial Ca^2+^ uptake also improves HF phenotype by reducing SR Ca^2+^ leak [40]. This highlights the pathophysiological role of the vicious cycle and mitochondrial ROS therein.

Ion channel remodelling in HF leads to APD prolongation and increased STV [22], creating a vulnerable arrhythmia substrate. APD prolongation then may promote further cellular Na^+^ and Ca^2+^ loading and CaMKII activation (Fig. 1). Inhibition of the upregulated I_NaL_, CaMKII and leaky RyRs all reduced APD prolongation and STV in HF (Fig. 2). In contrast, acute pharmacological induction of RyR leak by caffeine + isoproterenol did not change APD (Fig. 4). The more pronounced APD change by RyR leak in HF cardiomyocytes might reflect the effect of reduced repolarization reserve (downregulated K^+^ channels [46]) and altered balance between inward and outward ionic currents [23, 24]. In line with this, inhibition of I_Ks_ led to APD prolongation following β-adrenergic stimulation in rabbit (Fig. 3) and human [33] ventricular myocytes. Hamilton et al. [18] also showed that caffeine + isoproterenol but not isoproterenol alone increased mitoROS production. Moreover, we have shown a two-hit arrhythmia model in which hyperglycaemia-induced CaMKII activation and RyR leak alone did not change APD, but when repolarization reserve was reduced, a marked APD prolongation occurred [26]. Like with arrhythmia induction, arrhythmia termination may require two simultaneous targets. Such synergy was observed when either MitoTEMPOL or AIP was combined with GS-967 leading to a marked reduction in DADs (Figs. 5, 6). Then, in an inverse experimental setting, enhanced *I*_NaL_ prolonged APD (Fig. 7), increased [Ca^2+^]_i_, and induced EADs (Fig. 8). Multiple mechanisms can contribute to EADs, including spontaneous SR Ca^2+^ release and inward NCX, reopening of L-type Ca^2+^ channels (LTCC), and augmentation of I_NaL_, and all of these are modulated by [Ca^2+^]_i_ and CaMKII [30, 55]. CaMKII inhibition attenuated EADs (Fig. 8) and buffering [Ca^2+^]_i_ has been previously shown to abolish EADs induced by ATX-II [29]. Experimental [71] and computational modelling [13] studies mechanistically investigated the EAD mechanisms upon H_2_O_2_ treatment and showed that EADs emerge at slow pacing rates upon simultaneous activation of both LTCC and Na^+^ channels via ROS-dependent CaMKII activation (and alone, neither RyR nor *I*_NaL_ nor LTCC effects were sufficient to produce EADs). Intracellular Na^+^ loading induced by either ouabain [39] or ATX-II [34, 66] treatment has been shown to increase mitoROS and diastolic Ca^2+^-triggered arrhythmias. Here we showed that mitoROS also plays an important role in mediating EADs induced by the ATX-II-enhanced *I*_NaL_ (Fig. 8), which may reflect spatial and functional coupling between Na_V_1.5 channels and mitochondria [52]. Moreover, multiscale modelling of the mitochondria-SR microdomain showed that elevated ROS production increases [Ca^2+^]_*i*_ and arrhythmia propensity by stimulating RyRs and inhibiting SERCA [37]. Consistent with this, MitoTEMPOL pretreatment significantly prolonged EAD latency (Fig. 8) suggesting that the increase in ROS is an early response to [Na^+^]_*i*_ loading. Interestingly, inhibition of SR Ca^2+^ leak by dantrolene attenuated APD prolongation following ATX-II treatment (Fig. 7) and in HF (Fig. 2). The effects of dantrolene on APD (and EAD formation) might be explained by the attenuation of SR Ca^2+^ leak-induced CaMKII activity, changes in myocyte Na^+^ and Ca^2+^ loading, enhanced inward NCX, and late Ca^2+^ sparks, which can activate the vicious cycle and influence AP configuration [14]. Dantrolene also markedly attenuated APD prolongation, alternans, and spontaneous diastolic activities (i.e., DADs, sAPs) in hiPSC-CMs carrying SCN5A N406K mutation, highlighting the critical role of SR Ca^2+^ leak (and the activated vicious cycle) in these arrhythmias (Fig. 9).

Here, we aimed to preserve physiological regulation in our cellular experiments to uncover interactions within the feedback loops. However, this approach has limitations on quantifying the exact role that each component plays in the vicious cycle. Full inhibition of one key component may break the whole loop and have a marked arrhythmia reducing effect, like that seen in HF (Fig. 2). However, this approach may overestimate the individual contribution of one arm in the feedback loop. On the contrary, the APD shortening effect of dantrolene in HF (Fig. 2), ATX-II-induced long QT3 (Fig. 7), and SCN5A N406K (Fig. 9), and the antiarrhythmic effects of MitoTEMPOL and AIP in pharmacologically enhanced *I*_NaL_ (Fig. 5) and RyR leak (Fig. 8) clearly demonstrate the importance and strength of the vicious cycle. The interaction between [Na^+^]_i_ loading and ROS in promoting arrhythmias was found to be particularly strong, which then can lead to further RyR leak and CaMKII activation. In line with this, such synergy between multiple antiarrhythmic targets (e.g., inhibition of both Na^+^ channels and leaky RyRs) may contribute to the clinical benefit of flecainide [35] and ranolazine [51]. Future, mechanistic experiments (e.g., using permeabilized myocytes) could determine the quantitative relationship between [Na^+^]_*i*_ and mitochondrial ROS production at a given [Ca^2+^]_*i*_. Incorporating these data may help to constrain and improve computational models in the future, which then would allow more controlled analysis of different branches of the vicious cycle.

As discussed above, many components of the [Na^+^]_*i*_–[Ca^2+^]_*i*_–ROS-CaMKII-RyR leak vicious cycle signalling have already been shown; however, the strength of feedback interactions have not been previously investigated. Our conceptual novelty here is the identification of important *trans*-target effects beyond the *on*-target effects of the otherwise selective inhibitors. It may have important clinical implications suggesting that potentially a combination therapy targeting the major components of the arrhythmogenic vicious cycle described here can be synergistic and may provide substantial benefits in heart diseases by reducing cellular proarrhythmia. The use of combination therapy may also be advantageous by reducing the effective dose of each drug, thus reducing their adverse effects. Moreover, our data show that the most favourable drug target(s) may vary among heart diseases, and thus, personalized medicine approaches are required to identify the optimal drug combinations.

## Data Availability

All data and materials are available on reasonable request to the corresponding author.

## Abbreviations

AIP: Autocamtide-2-related inhibitory peptide
AP: Action potential
APD: Action potential duration
APD_90_: Action potential duration at 90% repolarization
ATX-II: Anemone toxin II
CaMKII: Ca^2^^+^/calmodulin-dependent kinase II
CaT: Ca^2^^+^ transient
CPVT: Catecholaminergic polymorphic ventricular tachycardia
DAD: Delayed afterdepolarization
EAD: Early afterdepolarization
hiPSC-CM: Human induced pluripotent stem cell-derived cardiomyocyte
HF: Heart failure
*I*_K1_: Inward rectifier K^+^ current
*I*_Ks_: Slow delayed rectifier K^+^ current
*I*_NaL_: Late Na^+^ current
ISO: Isoproterenol
LQT: Long QT
mitoROS: Mitochondrial reactive oxygen species
NCX: Na^+^/Ca^2^^+^ exchanger
ROS: Reactive oxygen species
RyR: Ryanodine receptor
sAP: Spontaneous action potential
sCaR: Spontaneous SR Ca^2^^+^ release
SR: Sarcoplasmic reticulum
STV: Short-term variability
WT: Wild-type
